# Imaging Features of Multiple Myeloma Extramedullary Lesions in the Liver with 18F-FDG PET/CT, Contrast-Enhanced CT and MRI

**DOI:** 10.3390/diagnostics9040179

**Published:** 2019-11-07

**Authors:** Dimitris Papamichail, Robert Hog, Hartmut Goldschmidt, Antonia Dimitrakopoulou-Strauss

**Affiliations:** 1 Clinical Cooperation Unit Nuclear Medicine, German Cancer Research Center (DKFZ), 69120 Heidelberg, Germany; 2 Internal Medicine V and National Center for Tumor Diseases (NCT), University Clinic Heidelberg, 69120 Heidelberg, Germany

**Keywords:** multiple myeloma, extramedullary, 18F-FDG PET/CT, MRI, contrast-enhanced CT

## Abstract

Ηepatic involvement in multiple myeloma is not common; nevertheless, it is associated with poorer outcome. Heterogeneous features have been described in few published reports so far. We present the imaging findings of PET/CT in comparison to those of MRI for two multiple myeloma (MM) patients, one with a liver lesion suspicious for myeloma metastasis on PET and one with multiple liver lesions suspicious for myeloma metastases on MRΙ. The subsequent ultrasound-guided needle biopsies confirmed the extramedullary spread of the disease in both patients. The first case exhibited a match in both functional imaging modalities (PET and MRI) but a mismatch of intense metabolic activity on ^18^F-fluorodeoxyglucose (18F-FDG) PET/CT and iso-attenuating liver parenchyma on contrast-enhanced CT. The second case showed a mismatch of signal elevation persistence on diffusion-weighted imaging (DWI) and physiologic 18F-FDG distribution in the liver parenchyma. These cases present different imaging features in MM lesions of the liver using PET/CT and MRI, reflecting the high disease heterogeneity in patients with MM and demonstrating that the use of both PET/CT and MRI may offer complementary information.

**Figure 1:**
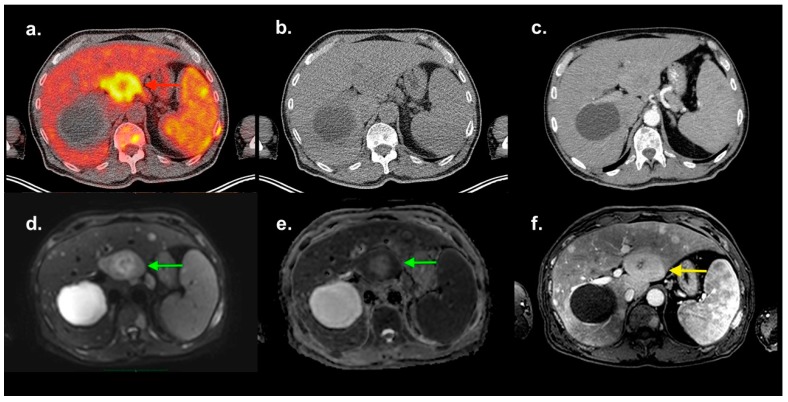
Newly diagnosed patient with multiple myeloma (MM, Typ IgA Kappa, Stage IIIA according to the Durie–Salmon staging system, ISS III), anemia and hepatosplenomegaly underwent an 18F-FDG PET/CT and a routine whole-body MRI investigation, as it is standard for all MM patients. The transaxial fused PET/CT image (**a**) shows a circumscribed lesion in the IVb segment of the liver (red arrow) with an SUVmax of 10.1, a functional volume of approx. 90 ml and a hypometabolic center. At the same level, a cystic liver lesion of 9 cm in diameter is delineated (without FDG-uptake). The FDG-avid lesion is not distinguishable in the low-dose CT (**b**). The contrast-enhanced diagnostic CT (**c**) shows a contrast uptake in the arterial phase nearly similar to the liver parenchyma, and a hypodense core demarks itself as in the PET/CT-image. Diffusion-weighted (DW **d**,**e**) MRI demonstrates an inhomogeneous abnormal diffusion restriction in the same area (green arrows), which can be clearly distinguished, showing an intermediate T1-signal intensity and a hyperintense core (yellow arrow) in the contrast-enhanced MRI (**f**). A biopsy of the lesion under sonographic control indicated plasma cell neoplasia. A diagnostic ultrasound of the liver was not performed before (no pre-described MM hepatic disease). The presence of extramedullary lesions at any time during the course of MM indicates a poor prognosis [1], and these patients may need to follow additional treatment strategies [2,3].

**Figure 2:**
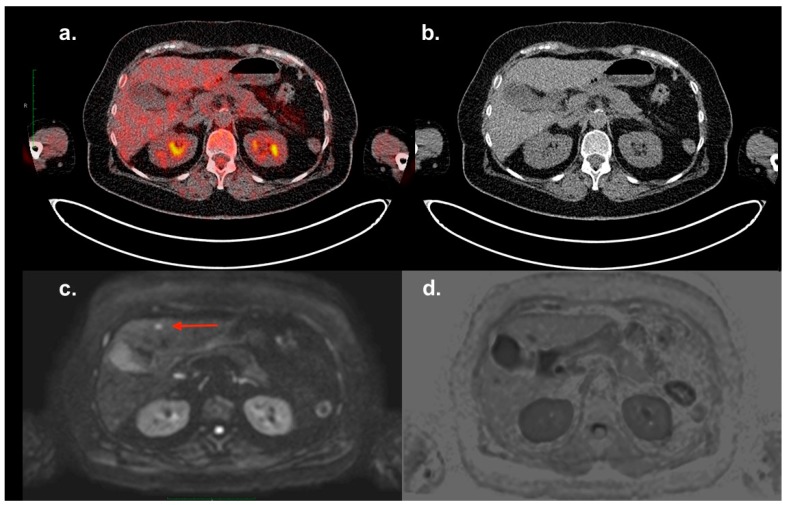
A patient with MM (Typ IgG lambda, Stage IA according to the Durie–Salmon staging system, ISS I) underwent an 18F-FDG PET/CT and a routine whole-body MRI investigation after radiation therapy of an extensive plasma cell infiltration in the sternum and three courses of induction therapy. No signs of further focal lesions were illustrated in previous CT and MRI investigations. A contrast-enhanced CT-scan was not performed in this case. The subsequent 18F-FDG PET/CT (**a**,**b**) did not demonstrate any focal lesion in the liver. A small lesion of 8 mm was delineated in Segment IVb, with an abnormal diffusion restriction in diffusion-weighted (DW, **c**) MRI (red arrow). The same lesion could not be distinguished in the T1-VIBE or T2-weighted sequences (**d**). A diagnostic ultrasound of the liver was not performed before (no pre-described hepatic disease), while the biopsy of the lesion under sonographic control indicated plasma cell neoplasia. Chen et al. reported a sensitivity of PET/CT nearly equivalent to that of MRI for the detection of extramedullary lesions; however, they added that in some areas, the use of both techniques could offer complementary information [4]. It must be noted that small lesions with a subcentimeter size may not be detectable on 18F-FDG PET, due to volume averaging in relation to the limited spatial resolution of the PET scanner [5]. This case report confirms the presumption of Chen et al. and reflects the high disease heterogeneity in patients with MM.
